# Anti-Tumor Effects of Second Generation β-Hydroxylase Inhibitors on Cholangiocarcinoma Development and Progression

**DOI:** 10.1371/journal.pone.0150336

**Published:** 2016-03-08

**Authors:** Chiung-Kuei Huang, Yoshifumi Iwagami, Arihiro Aihara, Waihong Chung, Suzanne de la Monte, John-Michael Thomas, Mark Olsen, Rolf Carlson, Tunan Yu, Xiaoqun Dong, Jack Wands

**Affiliations:** 1 Liver Research Center, Warren Alpert Medical School of Brown University and Rhode Island Hospital, Providence, Rhode Island, United States of America; 2 Department of Medical Chemistry, College of Pharmacy Glendale, Midwestern University, Glendale, Arizona, United States of America; 3 Department of Biomedical and Pharmaceutical Science, College of Pharmacy, The University of Rhode Island, Pharmacy Building, 7 Greenhouse Road, Kingston, Rhode Island, United States of America; University of Nebraska Medical Center, UNITED STATES

## Abstract

Cholangiocarcinoma (CCA) has a poor prognosis due to widespread intrahepatic spread. Aspartate β-hydroxylase (ASPH) is a transmembrane protein and catalyzes the hydroxylation of aspartyl and asparaginyl residues in calcium binding epidermal growth factor (cbEGF)-like domains of various proteins, including Notch receptors and ligands. ASPH is highly overexpressed (>95%) in human CCA tumors. We explored the molecular mechanisms by which ASPH mediated the CCA malignant phenotype and evaluated the potential of ASPH as a therapeutic target for CCA. The importance of expression and enzymatic activity of ASPH for CCA growth and progression was examined using shRNA “knockdown” and a mutant construct that reduced its catalytic activity. Second generation small molecule inhibitors (SMIs) of β-hydroxylase activity were developed and used to target ASPH *in vitro* and *in vivo*. Subcutaneous and intrahepatic xenograft rodent models were employed to determine anti-tumor effects on CCA growth and development. It was found that the enzymatic activity of ASPH was critical for mediating CCA progression, as well as inhibiting apoptosis. Mechanistically, ASPH overexpression promoted Notch activation and modulated CCA progression through a Notch1-dependent cyclin D1 pathway. Targeting ASPH with shRNAs or a SMI significantly suppressed CCA growth *in vivo*.

## Introduction

Cholangiocarcinomas (CCAs) are very aggressive tumors with high mortality due to early intrahepatic invasion and subsequent metastatic spread. The CCAs are classified into 3 subtypes as intrahepatic, extrahepatic, or hilar tumors [1]. The 5-year survival rate is 15% for localized disease and ~2% for advanced CCAs with distant metastases, respectively (American Cancer Society). Over the past several decades the incidence of CCAs has been rising in the United States [2]. Several potential risk factors have been identified to be associated with the development of CCAs, including age over 65, biliary stones, chronic infection with liver flukes, hepatitis B and C viruses, liver cirrhosis, and primary sclerosing cholangitis [1]. However, the underlying molecular mechanisms involved in CCA development and growth remain elusive.

To clarify the cellular factors responsible for initiation and progression of CCAs, several genetically engineered murine models have been developed, such as a double knockout of *SMAD4* and *PTEN* genes driven by an albumin promoter [3]; specific overexpression of the intracellular domain (ICN) of Notch1 driven by an albumin promoter [4]; a knockout of *NF2*[5]; overexpression of mutant *K-ras*^*G12D*^ and partial deletion of *p53* driven by an albumin promoter [6], as well as a direct knockout of *p53* driven by the *CK19* promoter [7]. Most of these genetic changes have been previously described in human tumors based on whole-exome sequencing of liver fluke-related and non-infection-related bile duct tumors[8]. Notch signaling has been critically involved in CCA’s pathogenesis since overexpression of the ICN in the liver led to CCA development in animal models [4].

Aspartate β-hydroxylase (ASPH) is a Type II transmembrane protein and belongs to the α-ketoglutarate-dependent dioxygenase family [9]. ASPH catalyzes the hydroxylation of aspartyl and asparaginyl residues located in the epidermal growth factor (EGF)-like domain of various proteins [10]. ASPH has been described to be overexpressed in placenta, as well as the embryo during different stages of development but has very low or negligible expression in adult tissues [11]. Interestingly, ASPH becomes re-expressed in tumors of liver, pancreas, lung and colon [12–14], suggesting that ASPH may be an oncogene involved in the transformation of normal cells to a malignant phenotype [15]. This hypothesis raises the possibility that targeting ASPH to reduce its level or activity may suppress tumor growth and inhibit cellular migration and invasion [9, 16].

Previous studies have shown that the transcriptional expression of ASPH is regulated through insulin -insulin-like growth factor 1 stimulated MAPK/ERK and PI3K/AKT pathways [17]. Importantly, in hepatocellular carcinoma (HCC), Notch signaling can be activated directly by ASPH upregulation [9] to promote tumor cell migration, invasion and metastases. Since activation of Notch signaling is proposed to play a key role in the pathogenesis of CCA, inhibition of this signaling pathway may produce anti-tumor effects [4]. Therefore, we hypothesized that overexpression of the ASPH protein could be a major factor for CCA development and progression; and targeting this enzyme with a potent second generation small molecule inhibitor (SMI) of β-hydroxylase activity that was developed by rational drug design based on the crystal structure of the catalytic site, would constitute a novel therapeutic approach for CCA.

## Materials and Methods

### Cell lines, animals, and reagents

Human cholangiocarcinoma cell lines, ETK1, H1, NEC, RBE, and SSP25 were provided by Dr. Munenori Enjoji at Kyushu University in Japan [16]. They were cultured in RPMI-1640 medium with 10% fetal bovine serum. BDE-Neu cells were provided by Dr. Alphonse E. Sirica at Virginia Commonwealth University [18]. BDE-Neu CL24 cells were a sub-clone previously established in our laboratory. OUMS-29, human hepatocyte cell line was provided by Dr. Hironori Koga at Kurume University in Japan [19]. HCC cell lines, BNLT3, Hep3B, HepG2, Huh7, and SkHep1 were purchased from American Type Culture Collection (Manassas, VA, USA). FOCUS was previously established in our laboratory [20]. HAK1A and HAK1B were kindly provided by Dr. Hironori Koga [21, 22]. Hepatocyte and HCC cell lines were maintained in DMEM medium supplement with 10% fetal bovine serum and 2 mM L-glutamine. All of the cell lines were cultured in a humidified incubator at 37°C with 5% CO_2_.

Six week old male nude mice (Charles River Laboratories) were used in animal studies. For the rat intrahepatic cholangiocarcinoma model, Fisher-344 male rats (Harlan Laboratories, Indianapolis, IN) weighing 150–200 g were employed. The intrahepatic inoculation and bile duct ligations were performed as previously described [18]. All *in vivo* procedures were approved by the Institutional Animal Care and Use Committee of Rhode Island Hospital.

Plasmids pLKO.1-shRNA-luciferase and pLKO.1-shRNA-ASPH were purchased from Sigma-Aldrich (St. Louis, MO). Plasmids of murine Notch1 reporter construct (12XCSL-DsRedExpressDL, #47683), constitutive active Notch1 (pCS2-Notch1ΔEMV-6MT, #41737), full length Notch1 (pCS2-Notch1 F.L.-6MT, #41728), and human intracellular domain of Notch1 (EF.hICN1.CMV.GFP, #17623) used in rescue experiments were purchased from Addgene (Cambridge, Massachusetts).

### Anti-tumor activity of a SMI in vivo

The animal protocol was approved by the Institutional Animal Care and Use Committee of Rhode Island Hospital. The H1 CCA cells (5×10^6^) were subcutaneously inoculated into 6 week-old male nude mice. After the tumor cells were implanted, the mice were evaluated 3 times per week. Once the tumor was visible or palpable, the mice were monitored and ant tumor growth was measured daily. The mice were also evaluated for body weight. Mice were euthanized once tumors reached 2000 mm^3^ (estimated by tumor volume = [(Width)2 X Length] / 2). Developed tumors with a volume of 50 mm^3^ were treated with the SMI (MO-I-1151). Mice were sacrificed with CO_2_ and tumors were removed for biological and biochemical assays at day 14.

Intrahepatic CCA models were established with BDE-Neu-CL#24 as previously described [18]. Basically, the rats were anesthetized with isoflurane and the abdomen was opened to expose the liver. 3x10^6^ BDE-Neu cells were inoculated into the rat liver. After injection, the rat bile duct was ligated and the BDE-Neu CCA cells were grown for 18 days. Buprenorphine was given at the dose of 0.03mg/kg subcutaneously before the surgery, four hours after and the next morning afterward for analgesia as necessary. There was one rat in control group died right after surgery. Other experimental rats were survived for whole experimental period. The rats were monitored daily. Since this tumor model was intrahepatic, it was not possible to monitor daily tumor size. Thus, the rats were evaluated for weight change, body condition, and signs of ascities accumulation. The rats were euthanized with CO2 and cervical dislocation. The tumors were dissected away from the adjacent liver to determine tumor volume and weight.

### Lentivirus production and infection of cholangiocarcinoma cells

HEK-293T cells were used to produce lentivirus that delivered shRNAs. In brief, HEK-293T cells were transfected with TransIT®-LT1 Reagent (Mirus Bio LLC.). Fifteen μg of lentivirus plasmids (pLKO.1-shRNA-luciferase and pLKO.1-shRNA-ASPH), 8.5 μg of psPAX2 (contains GAG, POL) and 5 μg of pMD2.G (contains VSVg) were used to produce virus in 10-cm dish. Culture medium containing virus was collected 48 hrs after transfection, passed through a 0.45 μm filter to remove debris, and added to the target cells. Infected cells were selected with puromycin at the concentration of 2 μg/ml for RBE and ETK-1 cells, and 3 μg/ml for H1 and SSP25 cells.

### Immunoblotting

Immunoblotting was performed as previously described [23]. In brief, 50–100 μg total protein was applied to the gels followed by the addition of first and second antibodies conjugated with horseradish peroxidase. Images were analyzed with Quantity One software (Bio-Rad).

### RNA preparation and RT-PCR

Cultured cells were collect with Trizol and mRNAs were extracted according to the manufacture’s protocol. The mRNAs (1 μg) were reverse transcribed to cDNA with iScript kit (Bio-Rad) as described in the instruction manual and cDNAs were used for assaying relative abundance of gene expression.

### Cell migration assay

The cell migration assay was performed using a Boyden Chamber transwell as previously described [16]. In brief, 200 μl of 10^5^ cells were prepared in serum free medium and seeded on top chamber; 600 μl culture medium containing 10% fetal bovine serum was applied on lower chamber to attract the cells. Cells were allowed to migrate for 16–24 hours, fixed with methanol, stained with 10% giemsa solution and counted.

### Cell growth assay

The CCA cell lines were seeded into 24-well plates and allowed to grow for 72 hrs as previously described [24]. Cells were trypsinized, suspended in culture medium, and counted with cytometer to determine the cell numbers.

### MTT assay

The CCA cells (2×10^3^) were seeded into 96-well plates and allowed to grow for 0, 1, 3, and 5 days. MTT solution was added to the wells and incubated for 60 minutes. The purple crystals were dissolved with DMSO and measured with an ELISA reader at 595 nm and normalized to the background at 690 nm.

### Colony formation in soft agar assay

The soft agar colony formation assay was performed as previously described [25, 26]. The CCA cells (2.5x10^3^) were mixed with 0.8% agar to form 0.4% agar mixture. The cells were cultured for 2 weeks, and formed colonies were stained with 10% giemsa solution and then counted.

### Cancer stem cell (CSC) sphere assay

The cancer stem cell sphere assay was performed as previously described [27]. In brief, 6×10^3^ CCA cells were seeded on low-attachment culture dish (10 cm) and incubated for 4 weeks with serum free DMEM containing 10 ng/ml EGF. The images of CSCs were taken using a light microscopic camera at 10× magnification.

### Statistical analysis

Data were described as mean ± SD. Student’s t-test was used to analyze the difference between two groups. A *p*-value of less than 0.05 was considered as statistically significant. To compare the means among ≥3 groups, one way analysis of variance (A.N.O.V.A) using the F distribution was employed.

## Results

### ASPH expression in CCA and HCC cell lines

ASPH has been previously shown to be upregulated in human CCA tumors by immunohistochemistry (IHC) using a monoclonal antibody (FB50); and more importantly, enhanced levels of ASPH were correlated with reduced survival rates as well as intrahepatic and lymph node metastases [13, 28]. Interestingly, ASPH is involved in HCC growth and progression principally by stimulating cell migration and invasion [9, 15]. Since hepatocytes and bile duct cells develop from a common precursor [29], it was of interest to evaluate the expression of ASPH in CCA and compare it to that in HCC cell lines. We observed that ASPH expression was even higher in CCA compared to HCC cell lines by Western blot analysis as shown in S1 Fig.

### ASPH expression promotes a more aggressive malignant phenotype dependent on its enzymatic activity

**Fig 1:**
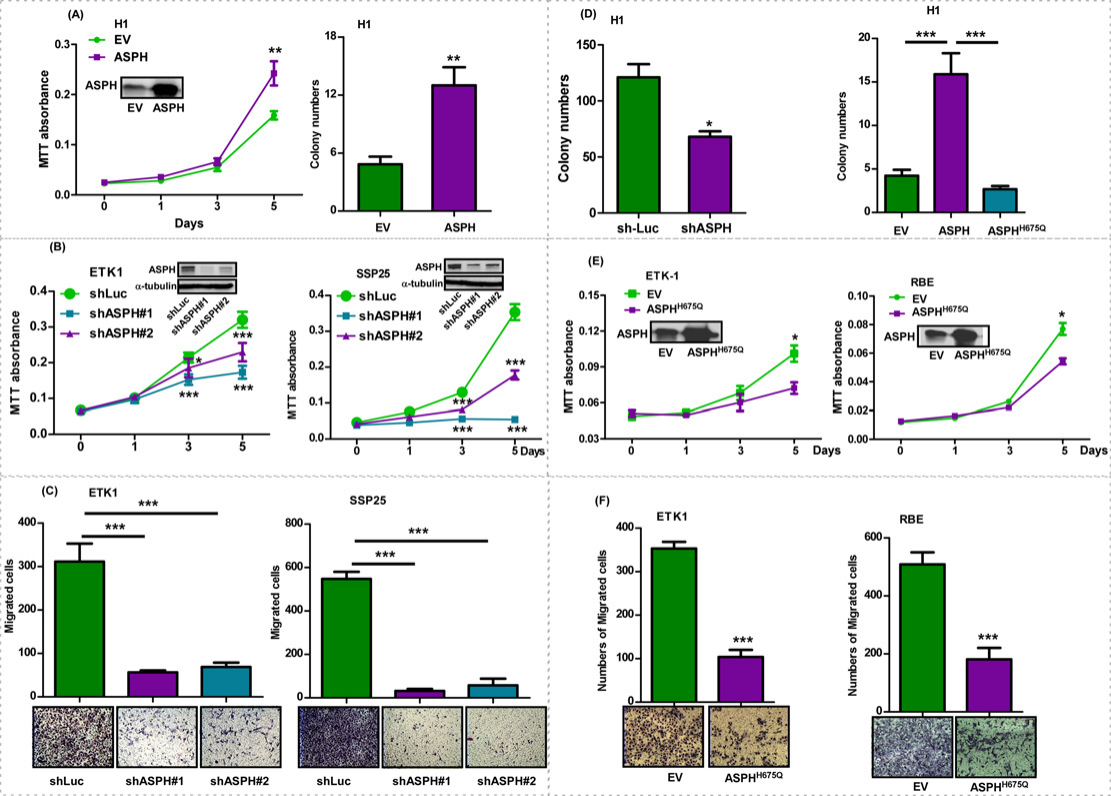
ASPH expression promoted malignant phenotypes in CCA cell lines. **(A)** MTT assay showing absorbance values measured at 0, 1, 3, and 5 days in H1 cells transfected with empty vector (EV) or ASPH. Right panel demonstrated colony formation in soft agar in H1-EV and H1-ASPH transfected cells. **(B)** A 5-day MTT and **(C)** 48 hr transwell migration assay were performed to determine cell proliferation and migration in ETK1 and SSP25 CCA cells infected with lentivirus containing shRNA-luciferase (shLuc), or shRNAs against ASPH (shASPH#1 and shASPH#2). Western blot analysis demonstrated decreased ASPH protein expression. **(D)** The number of colonies were measured in shLuc, shASPH, EV, ASPH, and ASPH^H675Q^ transfected H1 cells. **(E)** MTT absorbance and **(F)** migrated cell numbers were determined in ETK1 and RBE transfected with EV or ASPH^H675Q^. ***, *p* <0.001; **, *p* <0.01; *, *p* <0.05.

ASPH expression was either enhanced or inhibited in CCA cell lines by using a lentivirus transfection system. The knockdown efficiency of shRNAs against ASPH was evaluated in SSP25 cells. The shRNA #1 and #2 were chosen for further studies based on their inhibitory effects on ASPH protein and mRNA expression (S2A and S2B Fig). The overexpression of APSH, which was confirmed by immunoblotting in H1 CCA cells transfected with “wild type” (WT) ASPH construct, resulted in increased cell proliferation and anchorage-independent growth as measured by a MTT assay and colony formation in soft agar (Fig 1A). Knockdown of ASPH using shRNAs was performed in ETK1, H1, RBE, and SSP25 CCA cells. The effects of ASPH downregulation on the malignant phenotype were determined by cell viability, proliferation, and migration assays (Fig 1B and 1C; S3A and S3B Fig). Knockdown of ASPH in H1 cells significantly inhibited anchorage-independent cell growth (Fig 1D), as an additional index of attenuating the transformed phenotype.

The enzymatic activity of ASPH was proposed to be essential for modulating the biologic function of certain proteins such as Notch receptors and ligands that contain EGF-like domains for β-hydroxylation [30, 31]. A mutant ASPH (ASPH^H675Q^) construct was constructed and employed to test whether the enzymatic activity of ASPH was pivotal in modifying the CCA phenotype. In this context, a histidine was replaced by glutamine at the enzymatically functional site at amino acid residue 675 and it reduced ASPH enzymatic activity by 80% [32]. Overexpression of ASPH increased the formation of colonies in soft agar, whereas overexpression of ASPH^H675Q^ reduced colony formation and altered the phenotype of CCA cells (Fig 1D, right panel), indicating that loss of enzymatic activity may block its function. Furthermore, we overexpressed ASPH^H675Q^ in ETK1, NEC, RBE, and SSP25 cell lines with relatively higher levels of endogenous ASPH expression and subsequently evaluated the effects on the CCA malignant phenotype. The overexpression of mutant ASPH^H675Q^ significantly reduced cell viability and migration (Fig 1E and 1F, S3C and S3D Fig). These observations suggest the importance of ASPH enzymatic activity in generating a malignant phenotype.

### ASPH activates Notch signaling and downstream transcriptional activity

**Fig 2:**
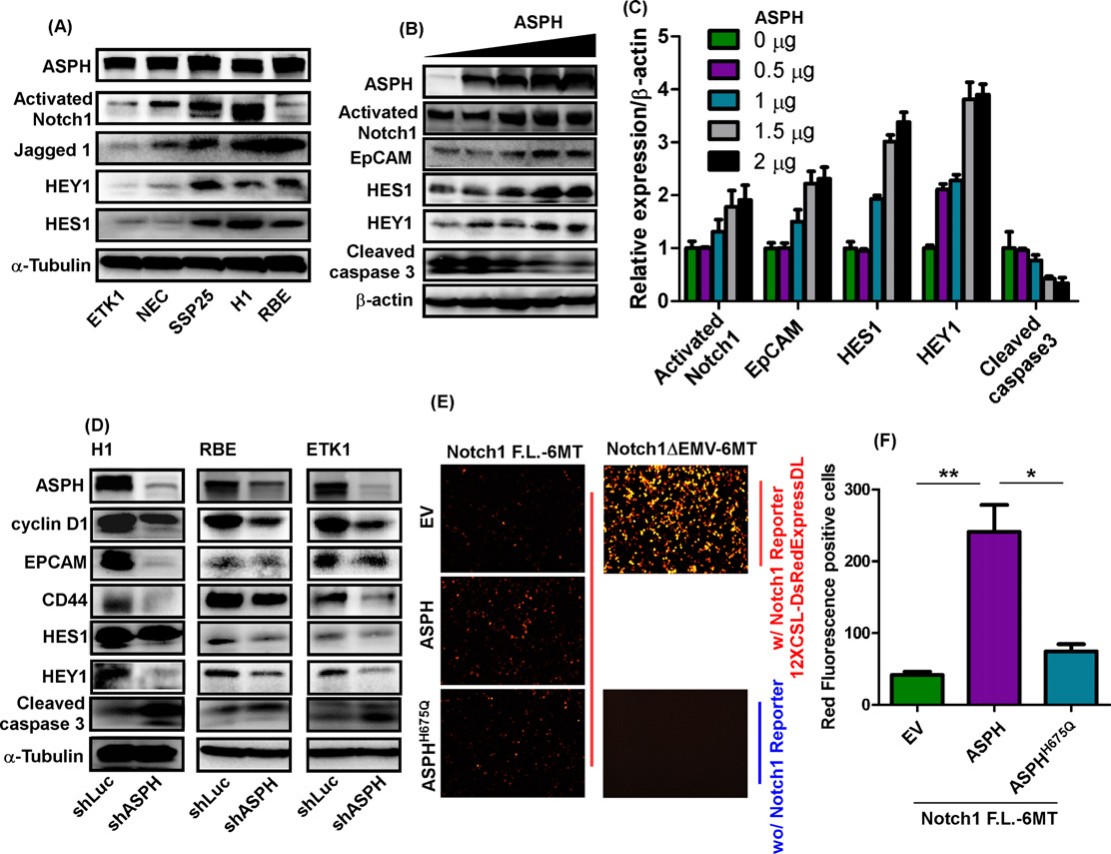
ASPH modulated Notch signaling in CCA cells. **(A)** Protein expression levels of ASPH, activated Notch1, JAG1, HEY1, and HES1 in 5 CCA cell lines. **(B)** ASPH, cyclin D1, EpCAM, HES1, HEY1, and cleaved caspase-3 in HEK293 cells transfected with WT-ASPH plasmid at concentrations of 0, 0.5, 1, 1.5, 2, and 2.5 μg. **(C)** Semi-quantitation of immunoblotting results depicted in (**B**). **(D)** Expression levels of ASPH, cyclin D1, EpCAM, CD44, HES1, HEY1, and cleaved caspase-3 in H1, RBE, and ETK1 cells infected with lentivirus containing either shLuc or shASPH. **(E)** pCS2-Notch1-full-length-6MT (pCS2-Notch1-F.L.-6MT), EV, ASPH, ASPH^H675Q^, and 12XCSL-DsRedExpressDL (Notch reporter) were co-transfected into HEK293 cells and image of red fluorescence was quantified under a fluorescence microscope. (F) Quantitation of red fluorescence signals are presented. Constitutive active Notch1 (pCS2-Notch1-ΔEMV-6MT) was used as a positive control. Transfection of Notch reporter construct alone was used as a negative control.

The Notch signaling pathway was variably activated in 5 CCA cell lines with high endogenous expression of ASPH as demonstrated by the generation of activated Notch1 ICN, high level expression of Jagged1 (JAG1) and upregulation of downstream target genes (e.g., HEY1 and HES1) (Fig 2A). We focused on Notch signaling because direct overexpression of the Notch1 ICN initiated CCA tumor growth in the murine liver without known genetic alterations [4]. To clarify the mechanisms by which ASPH influences Notch signaling, WT-ASPH was transiently and dose-dependently transfected into HEK293 cells. ASPH expression was confirmed by Western blot analysis followed by generation of enhanced Notch signaling characterized by upregulation of HES1, HEY1 and CSC marker EpCAM. In contrast, ASPH induced downregulation of cleaved caspase-3, a major driver of apoptosis (Fig 2B and 2C).

Knockdown of ASPH using shRNAs in H1, RBE, and ETK1 cells reduced the expression of ASPH, cyclin D1, EpCAM, CD44, HEY1, and HES1; but increased the level of cleaved caspase-3, which contributed to an inhibition of cell growth and proliferation as shown in Fig 2D. The HES1 mRNA expression was examined to determine whether Notch transcriptional activity was present or not. Knockdown of ASPH in H1 and RBE CCA cells significantly inhibited mRNA expression of HES1 (S4A and S4B Fig), suggesting that Notch transcriptional activity was regulated by the level of ASPH expression. To further confirm this finding, full length Notch1 (pCS2-Notch1-F.L.-6MT) and constitutive active Notch1 mutant (pCS2-Notch1-ΔEMV-6MT) accompanied with Notch1 Reporter (12XCSL-DsRedExpressDL) constructs were co-transfected with empty vector (EV), WT-ASPH and mutant ASPH^H675Q^ constructs into HEK293 cells. As shown in Fig 2E and 2F, constitutive active Notch1 substantially increased Notch1 reporter expression as shown by red fluorescence (positive control). Full length Notch1 marginally promoted Notch1 reporter expression; and the induction of Notch1 reporter activity was much higher following co-transfection of ASPH. However, this activity was reduced when the ASPH^H675Q^ mutant construct with 80% reduced enzymatic activity was co-transfected with full length Notch1, suggesting that ASPH enhanced the transcriptional activity of Notch signaling and the enzymatic activity of ASPH was required for this biologic activity.

### ASPH promotes CCA cell proliferation through Notch1-mediated cyclin D1 expression

**Fig 3:**
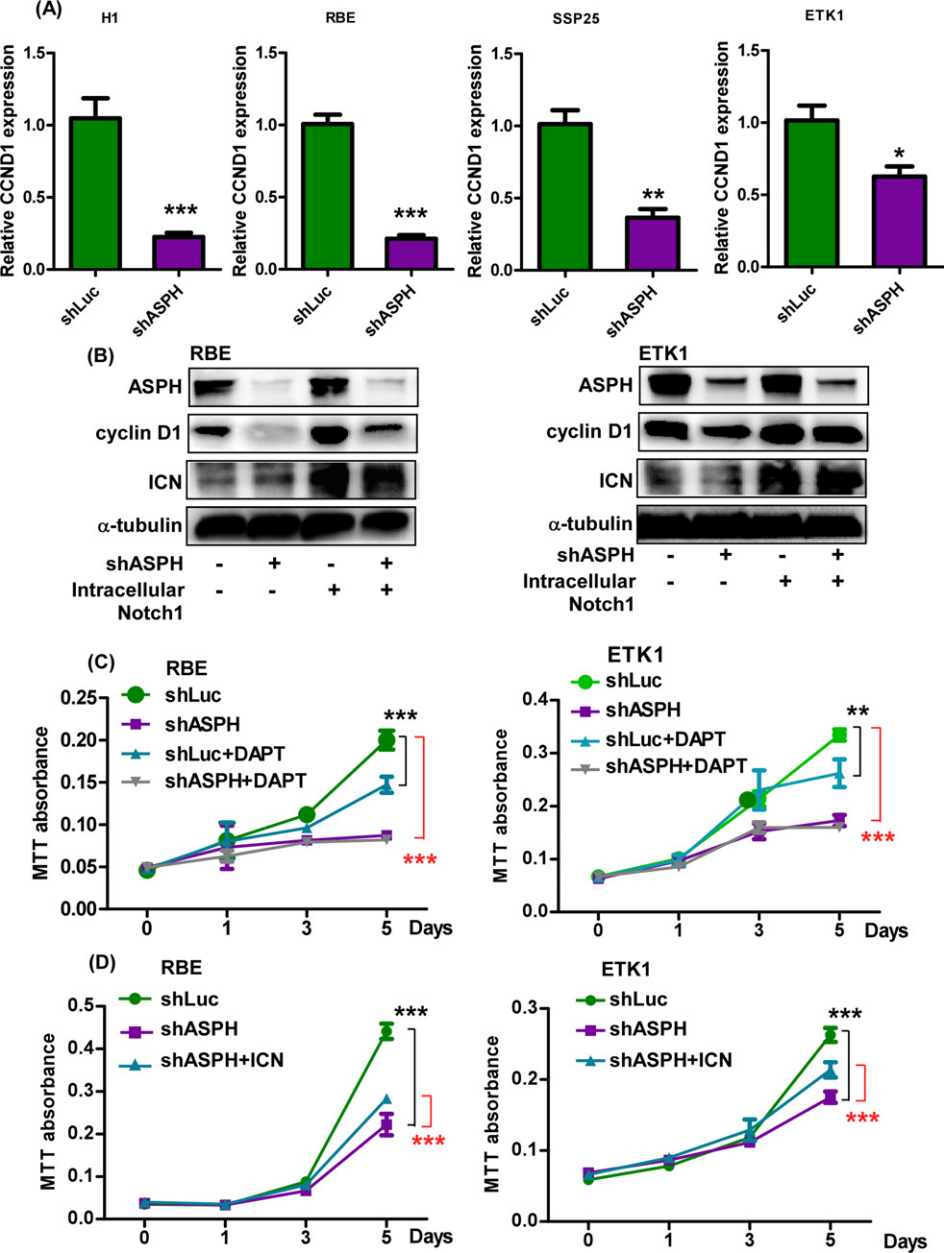
ASPH upregulated the expression of cyclin D1 expression and accelerated cell proliferation. **(A)** Relative CCND1 mRNA expression in H1, RBE, SSP25, and ETK1 cells infected with lentivirus containing shLuc or shASPH. **(B)** Immunoblotting of ASPH, cyclin D1, and intracellular domain of Notch1 (ICN) in RBE-shLuc, RBE-shASPH, ETK1-shLuc, and ETK1-shASPH transfected cells with or without overexpression of ICN. **(C)** Relative cell proliferation in RBE-shLuc, RBE-shASPH, ETK1-shLuc, and ETK1-shASPH in the presence or absence of 10 μM of the γ-secretase inhibitor DAPT using the MTT assay. **(D)** Relative cell proliferation rate in RBE-shLuc, RBE-shASPH, RBE-shASPH transfected cells overexpressing ICN. ***, *p* <0.001; **, *p* <0.01; *, *p* <0.05.

Cyclin D1 as a member of the cyclin protein family involved in cell cycle regulation has been demonstrated to be amplified and overexpressed in CCA[33]. We proposed that its expression is regulated by Notch1 signaling [34], which is important for generation of a CCA malignant phenotype. The expression level of cyclin D1 was measured in shRNA-ASPH CCA cell lines. Knockdown of ASPH substantially reduced cyclin D1 expression. Furthermore, overexpression of the Notch1 ICN partially restored cyclin D1 level (Fig 3A and 3B), suggesting that cyclin D1 acts as a downstream effector to promote CCA cell proliferation. To compare the inhibitory efficiency between ASPH knockdown and Notch signaling inhibition in CCA, DAPT (N-[(3,5-Difluorophenyl)acetyl]-L-alanyl-2-phenyl]glycine-1,1-dimethylethyl ester), a γ-secretase inhibitor, was employed. We observed that DAPT, a potent inhibitor of Notch signaling, reduced cell growth (to a much less extent than ASPH-shRNAs) but had no effect on migration of CCA cell lines (Fig 3C; S5A and S5B Fig). Furthermore, the inhibitory effect of shRNA-ASPH on CCA cell growth was partially restored by overexpression of the ICN as well as cyclin D1 (Fig 3D and S5C Fig).

### A SMI of ASPH suppresses the CCA malignant phenotype

**Fig 4:**
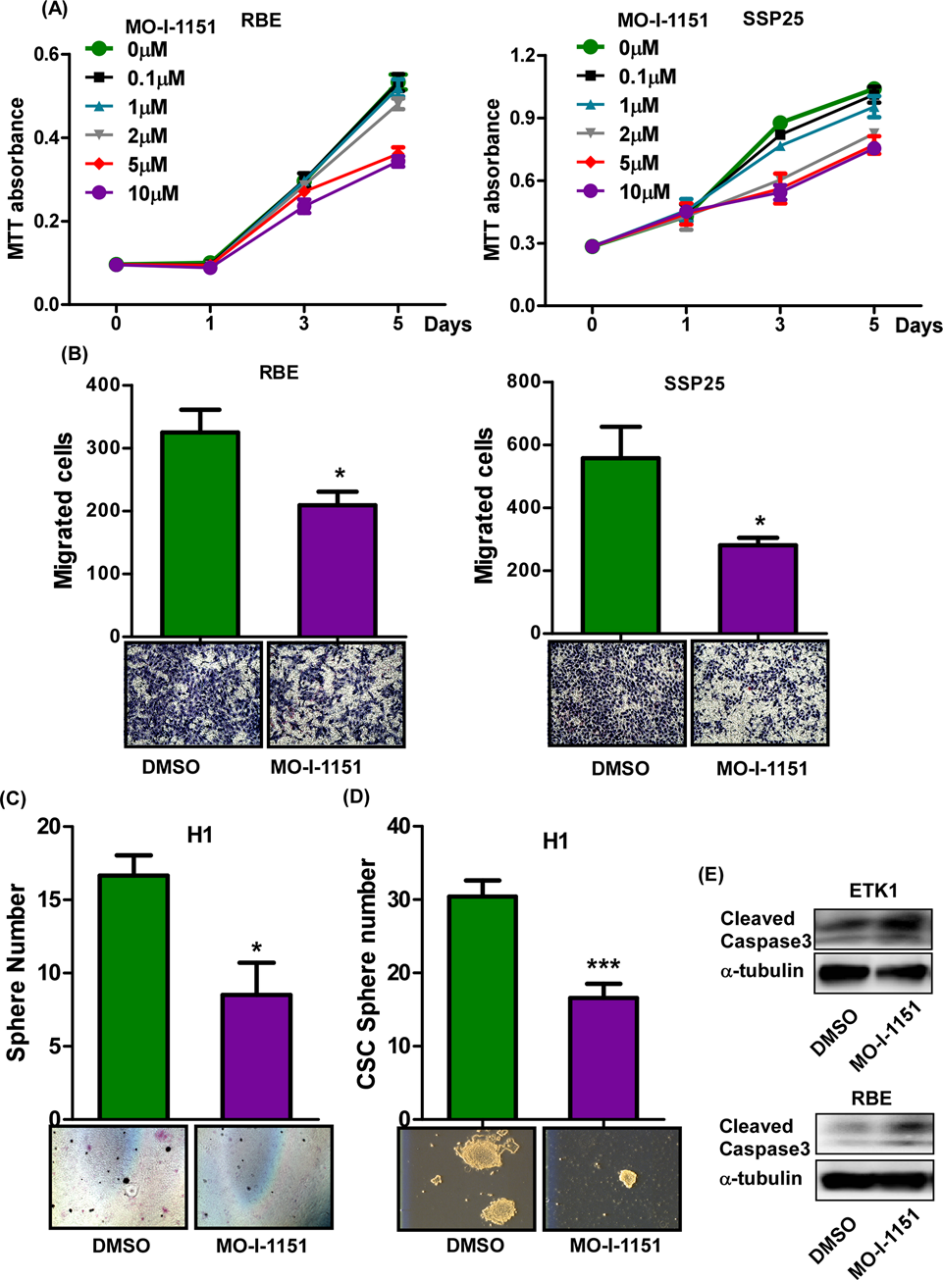
Functional characterization of a SMI, MO-I-1151 on CCA phenotype. Relative cell proliferation rates were measured using MTT assay at day 0, 1, 3, and 5 in RBE and SSP25 cells **(A). (B)** Migrated cell numbers were determined in RBE and SSP25 in the presence or absence of 5 μM MO-I-1151. **(C)** Colony formation in soft agar was determined in H1 cells treated with or without MO-I-1151 at 5 μM. **(D)** Formation of CSC spheres were determined in H1 cells treated with DMSO or 5 μM MO-I-1151. (**E**) MO-I-1151 also increased caspase 3 cleavage in ETK1 and RBE cells. ***, *p* <0.001; *, *p* <0.05.

The facts that ASPH is highly expressed in human CCA tumors/cell lines; overexpression or knockdown of ASPH promotes or inhibits CCA malignant phenotype, respectively, lead us to explore if inhibition of the enzymatic activity by SMIs would alter cellular behavior of CCA. We had previously identified 1st generation ASPH inhibitors, such as MO-I-1100 [9]. We evaluated the efficacy of this compound with the structure depicted in S6A Fig and found that MO-I-1100 suppressed H1 cell proliferation at 1 μM but had no significantly biological effects on the other human CCA cell lines (data not shown). Therefore, the side groups of the parent molecule were modified (S6B Fig) in an attempt to improve its potency and solubility and then to determine its effects on cell viability as shown in S6C Fig. Three newly synthesized compounds, MO-I-1144, MO-I-1150, and MO-I-1151 (S6B Fig) were tested and all the 3 compounds demonstrated more potent inhibitory effects compared to the parent compound by 10–50 fold (S6C Fig). Among them, MO-I-1151 consistently produced better suppression of cell viability (S7 Fig). Thus, MO-I-1151 was selected for *in vivo* studies since it showed dose-dependent inhibition of CCA cell proliferation and migration (Fig 4A–4D and S8A Fig). MO-I-1151 consistently suppressed colony formation in soft agar and CSC sphere formation in H1 cells, and enhanced caspase-3 cleavage (Fig 4E).

### Inhibiting ASPH expression and enzymatic activity alters CCA tumor growth and progression in vivo

**Fig 5:**
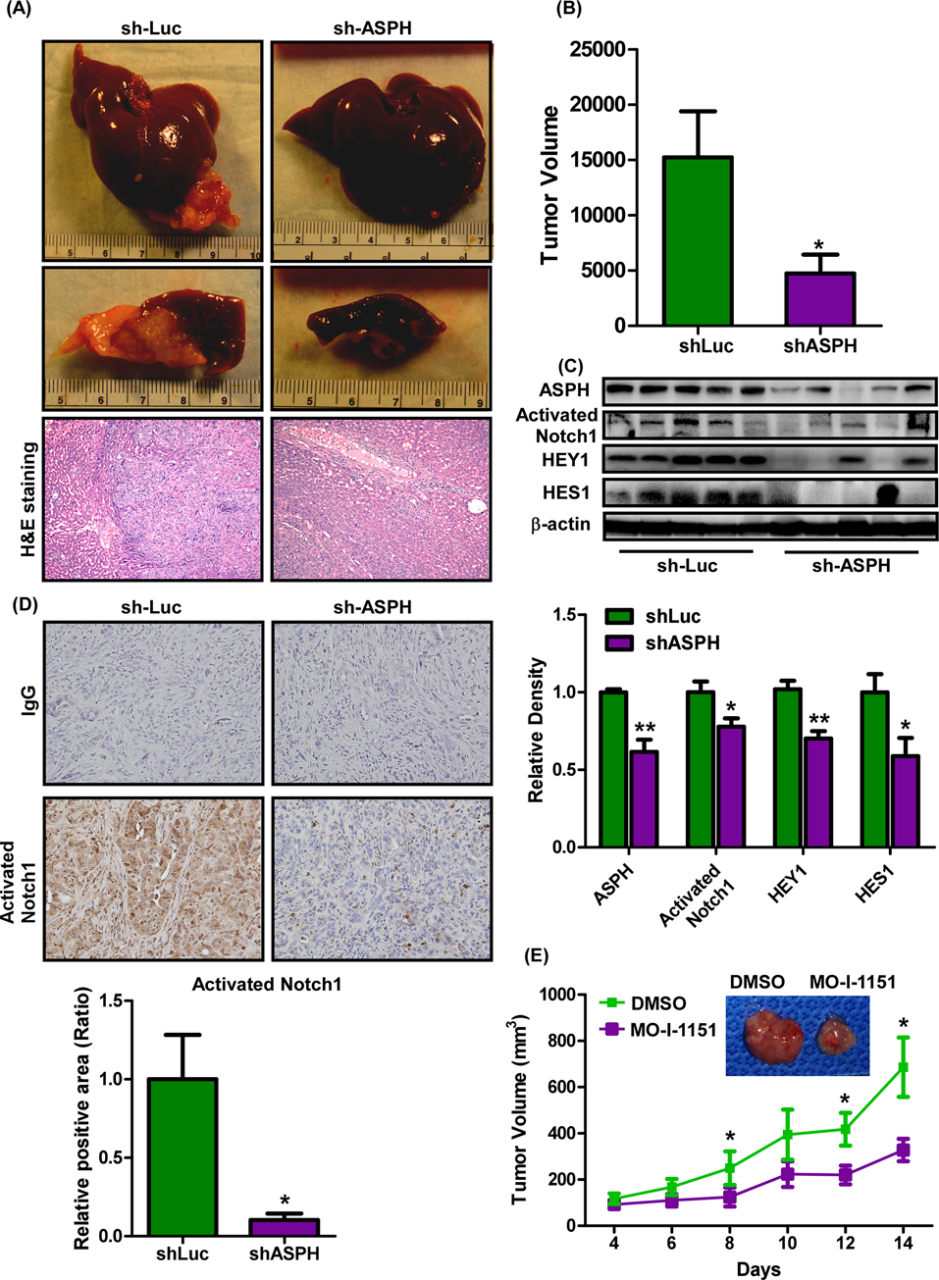
Anti-tumor effects of targeting ASPH in a rat intrahepatic CCA model of intrahepatic growth as well as subcutaneous tumor growth in nude mice xenograft with a human H1 CCA cell line. **(A)** Gross morphology and histology (H&E staining) of rat livers inoculated with BDE-Neu-CL#24-shRNA-luciferase (shLuc) or BDE-Neu-CL#24-shRNA-ASPH (shASPH). **(B)** Tumor volume in rat livers of BDE-Neu cell clone (#24) generated intrahepatic CCA tumors. **(C)** (Upper) Expression of ASPH, activated Notch1, HES1, and HEY1 in shLuc and shASPH treated in BDE-Neu-CL#24 generated tumors. (Lower) Relative expression abundance was shown by the density measurements. (**D**) (Upper) Representative IHC images of activated Notch1 in shLuc and shASPH treated rat intrahepatic CCA. (Lower) Number of positive nuclei containing an activated Notch1 signal was calculated. **(E)** H1 xenograft tumor growth rate and progression was determined in nude mice receiving DMSO or MO-I-1151 treatment every other day at 25 mg/kg. **, *p* <0.01; *, *p* <0.05.

Preclinical CCA animal models were generated to confirm the antitumor effects of inhibiting ASPH function. The rat BDE-Neu cell line was employed. This cell line has been derived from a normal rat bile duct epithelial (BDE) cells transformed with the Her2/Neu proto-oncogene to generate CCA tumors in rat’s liver [18]. BDE-Neu cells were stably infected with lentivirus containing shRNA-luciferase (sh-Luc) and shRNA-ASPH (sh-ASPH) and intrahepatically inoculated into rats following bile duct ligation. Intrahepatic inoculated BDE-Neu cells transfected with sh-Luc served as a control and demonstrated growth of large tumors around the bile ducts, but BDE-Neu cells with stably transfected sh-ASPH had a substantial reduction in tumor growth (Fig 5A and 5B). There was no change in body weight (S9A Fig). The expression levels of ASPH, activated Notch1, HES1, and HEY1 were examined in the rat CCA tumors to evaluate the knockdown efficacy on ASPH levels and functions as measured by inhibition of ASPH-mediated Notch signaling. The levels of ASPH, activated Notch1, HES1, and HEY1 were downregulated in sh-ASPH expressing rat CCA tumors (Fig 5C). The IHC staining results revealed that activated Notch1 signaling was very robust in sh-Luc treated tumor tissue as compared to sh-ASPH treated cells (Fig 5D), suggesting that targeting ASPH with shRNAs substantially inhibits Notch signaling and blunts CCA growth. Based on these results, we evaluated the anti-tumor effects of MO-I-1151 using human H1 CCA cells to generate subcutaneous tumors in nude mice. MO-I-1151 was found to significantly suppress CCA tumor growth and progression without affecting body weight (S9B Fig). MO-1151 treatment inhibited generation of activated Notch1 and Hey1 overexpression in the H1 grown subcutaneous tumors (S9C Fig). In these experiments when CCAs reached a size of approximately 50 mm^3^, MO-I-1151 (25 mg/kg) was administered intraperitoneally every other day for 14 days. We observed substantially delayed tumor growth and progression (Fig 5E).

## Discussion

ASPH is expressed during embryonic development[11] whereas it has very low level or negligible expression in adult tissues [13]. Notably, ASPH re-emerges and becomes highly expressed in various tumors and is particularly prominent in CCAs with a positive rate of >95%, suggesting a possible oncogenic role for this molecule during malignant transformation of bile duct cells. ASPH can promote a more malignant phenotype following transfection but the molecular mechanisms have not been clarified [14, 15]. ASPH directly interacts with Notch1 and JAG1 in HCC cell lines and thus activates Notch signaling [35, 36]. Our recent study using one of the first generation SMIs of ASPH enzymatic activity led to downregulation of Notch signaling [9, 14].

ASPH overexpression has been indicated to act as a direct activator of the Notch signaling cascade and promotes CCA growth and progression by generating a more aggressive malignant phenotype. Consistently, ASPH expression correlated with the activation of Notch downstream targets, such as HES1 and HEY1. Under these circumstances, ASPH was found to regulate the transcriptional activity of Notch. We have observed that ASPH modulated the transcriptional activity of full-length Notch1 but not constitutively active Notch1 which lacks the extracellular domain that contains the multiple EGF-like motifs required for β-hydroxylation. In addition, a Notch downstream target, cyclin D1, was transcriptionally modulated by Notch signaling [33] in CCA, suggesting it could be an important effector in regulating cell cycle. In this context, we observed that ASPH upregulated cyclin D1 expression in a Notch-dependent manner. Our results identified a novel molecular mechanism of “ASPH→Notch→cyclin D1” as a driver of CCA growth and progression.

DAPT, a γ–secretase inhibitor, has been previously shown to inhibit CCA progression based on the rationale that specific overexpression of ICN in mouse liver initiated CCA development, and inhibition of γ–secretase would suppress ICN production [4]. Although targeting Notch signaling might be effective in treating CCAs, the adverse effects on the gastrointestinal tract [37, 38] are problematic; therefore, ASPH may be a more specific target to alter Notch activity since MO-I-1151 significantly inhibits CCA cell proliferation, migration, invasion, colony formation and CSC generated spheroids by inhibition of its β-hydroxylase activity, which is more potent and efficient than DAPT.

ASPH-modulated cyclin D1 expression was Notch dependent, however, restoration of ICN levels in sh-ASPH treated CCA cells could not completely restore their growth characteristics. DAPT treatment did not reach the magnitude of Notch suppression caused by sh-ASPH in CCA cell lines. Furthermore, sh-ASPH treated CCAs exhibited a substantial reduction in cell migration, but DAPT failed to influence this phenotype. There are several potential candidate proteins containing cbEGF-like domains that participate in cancer metastasis, such as latent transforming growth factor-β (TGFβ) binding proteins (LTBPs), growth arrest specific 6 (GAS6) and CD97. TGFβ has been extensively investigated regarding epithelial-mesenchymal-transition (EMT) and is a therapeutic target to reduce tumor metastases[39]. GAS6 and related AXL receptor tyrosine kinase have been shown to be involved in metastasis of renal cancer and osteosarcoma [40, 41]. CD97 is a multifunctional leukocyte receptor, with a role in invasion and prognosis [42]. To clarify the mechanisms of ASPH-mediated CCA migration, future studies will be required to distinguish which proteins containing EGF-like domains are involved in ASPH generated metastatic spread of tumor cells.

In addition to ASPH mediated increase in CCA proliferation and migration, CSC markers (CD44 and EpCAM) were enhanced whereas apoptosis was inhibited by ASPH overexpression. Interestingly, apoptosis was stimulated through increasing caspase-3 cleavage and reducing enzymatic activity via exposure to a SMI of ASPH’s enzymatic activity. Targeting ASPH may suppress CSC generation and promote apoptosis, which is another key event to control tumor cell proliferation and progression. We observed that both shRNA-ASPH and MO-I-1151-treated CCA cells induced the upregulation of cleaved caspase-3.

CSCs properties were altered upon shRNA-ASPH treatment in CCA cells. CSCs represent a small population of cells within tumors capable of extensive self-renewal and acting as a driving force for cancer progression. The existence of CSCs has emerged as a cause for therapeutic resistance and clinical relapse. Although there is controversy regarding the properties of CSCs [43]; such cells following purification by antibodies directed against cell surface markers become more resistant to chemotherapy and targeted therapy, [44, 45] which further supports their role in tumor progression and metastasis. Notch signaling has been investigated extensively in CSCs biology [46, 47], and overexpression of ICN in hepatocytes initiates CCA development, implying that Notch signaling is critical for ASPH-induced expression of CSC markers. In this study, we demonstrated that ASPH positively modulated transcriptional activity of Notch signaling using a Notch reporter assay. Furthermore, Notch signaling was important in ASPH-mediated CSCs sphere formation. However, we did not observe any effects of the γ-secretase inhibitor DAPT on CSC sphere numbers. In contrast, a SMI of ASPH enzymatic activity inhibited CSC sphere formation of CCA cell lines in a dose-dependent manner. EpCAM and CD44 expression was used to identify this CSC subpopulation in CCAs. Our study provides evidence that targeting ASPH with shRNAs or 2nd generation SMIs could substantially not only suppress CCA growth and migration *in vitro* but also reduce CCA progression *in vivo* in a rat model of intrahepatic growth as well as in a human CCA xenograft murine model.

Taken together, ASPH modulates CCA progression, and links upstream growth factor signaling to downstream Notch cascade activation[9]. Better understanding of the signaling pathways important in the pathogenesis of CCA may provide new molecular targets for therapy of this progressive and usually fatal disease.

## Supporting Information

S1 Fig Expression of ASPH in HCC and CCA cell lines using Western blot analysis. ASPH was undetectable in human hepatocytes. Variable levels of ASPH expression were detected in HCC cell lines. High ASPH expression was observed in all 5 CCA cell lines. β-actin served as the protein loading control.
(TIF)

S2 Fig Characterization of shRNA-ASPH in SSP25 cells. **(A)** Immunoblotting results of ASPH expression. SSP25 cells were infected with lentivirus containing shRNA-luciferase (shLuc), shRNA-ASPH (shASPH) #0, #1, #2, #3, and #4. **(B)** Relative ASPH mRNA expression was determined as indicated.
(TIF)

S3 Fig Effects of ASPH knockdown on CCA malignant phenotype. **(A)** MTT assay results were determined at days 0, 1, 3, and 5 in H1 and in RBE cells infected with lentivirus containing shLuc or shASPH. **(B)** Cell proliferation as determined by cell counting for H1, RBE, ETK1, and SSP25 CCA cells infected with lentivirus containing shLuc or shASPH. **(C)** MTT assay results were measured in NEC cells transfected with empty vector (EV) or mutant ASPH^H675Q^ with 80% reduced enzymatic activity. **(D)** Migrated cell numbers were measured in NEC and SSP25 cells transfected with EV or ASPH^H675Q^. ***, *p*-value <0.001; **, *p*-value <0.01; *, *p*-value <0.05.
(TIF)

S4 Fig Effects of ASPH knockdown on Notch downstream target gene expression. **(A)** Relative ASPH mRNA expression was determined in H1 and RBE CCAs infected with lentivirus containing shLuc or shASPH. **(B)** Relative HES1 mRNA expression was shown as indicated. ***, *p*-value <0.001; **, *p*-value <0.01; *, *p*-value <0.05.
(TIF)

S5 Fig Effects of Notch/cyclinD1 signaling on CCAs cellular behaviors. **(A)** MTT assay results obtained in H1 and SSP25 cells with shLuc or shASPH in the presence or absence of 10 μM γ-Secretase inhibitor (DAPT). **(B)** Migrated cell numbers were measured in RBE, SSP25, and ETK1 cells as indicated. DAPT had little effect on CCA viability and migration. (C) Relative cell growth rate was determined in H1-shLuc, H1-shASPH, RBE-shLuc, and RBE-shASPH in the presence or absence of cyclin D1 (CCND1) as indicated. *, *p*-value <0.05; ***, *p*-value <0.001.
(TIF)

S6 Fig Characterization of the 2^nd^ generation SMI. **(A)** Structure of the 2nd generation SMI compound MO-I-1151. **(B)** Structures of representative 2nd generation SMIs of β-hydroxylase activity. **(C)** An analysis of their effect on cell viability. 2^nd^ generation SMIs were 10–50 times more potent than the parent MO-I-1100 compound and MO-I-1151 was selected for further studies based on its strong inhibitory effect.
(TIF)

S7 Fig Role of MO-1144, MO-1150, and MO-1151 in inhibiting CCA cell proliferation. Relative cell growth curves were determined in RBE and ETK1 cells in the presence of MO-I-1144, MO-I-1150, and MO-I-1151, respectively, at the indicated concentrations. MO-I-1151 was found to be the most active and potent.
(TIF)

S8 Fig Effects of MO-1151 on migration and CSC sphere formation. **(A)** Migrated cell numbers were measured in BDE-Neu cells treated with DMSO or 10 μM MO-I-1151. **(B)** CSC sphere numbers were determined in H1 cells treated with MO-I-1151 at indicated concentrations or 10 μM DAPT. (**C**) 12XCSL-DsRedExpressDL (notch reporter) was transfected in RBE CCA cells and then the cells were treated with MO-I-1151 as indicated concentrations. Red fluorescence positive cells were quantified as an index for notch transcriptional activity. ***, *p* <0.001; *, *p* <0.05.
(TIF)

S9 Fig The impact of inhibiting ASPH mediated signaling *in vivo*. (A) The body weight of rats inoculated with BDE-Neu-shLuc and BDE-Neu-shASPH. (B) The body weight of the mice subcutaneously injected with H1 cells and challenged with vehicle (DMSO) or MO-I-1151 showing no change over 14 days. (C) Immunoblot results of activated Notch1, Hey1, and α-tubulin were determined in H1 tumors (n = 5) treated with vehicle (DMSO) or MO-I-1151. (D) Densitometry of the immunoblots (*p<0.05; **p<0.01).
(TIF)
