# Supplementary figures and images for: Anti-Tumor Effects of Second Generation β-Hydroxylase Inhibitors on Cholangiocarcinoma Development and Progression
Source: PLoS One. 2016 Mar 8;11(3):e0150336. doi: 10.1371/journal.pone.0150336 (PMC4783022; doi:10.1371/journal.pone.0150336)

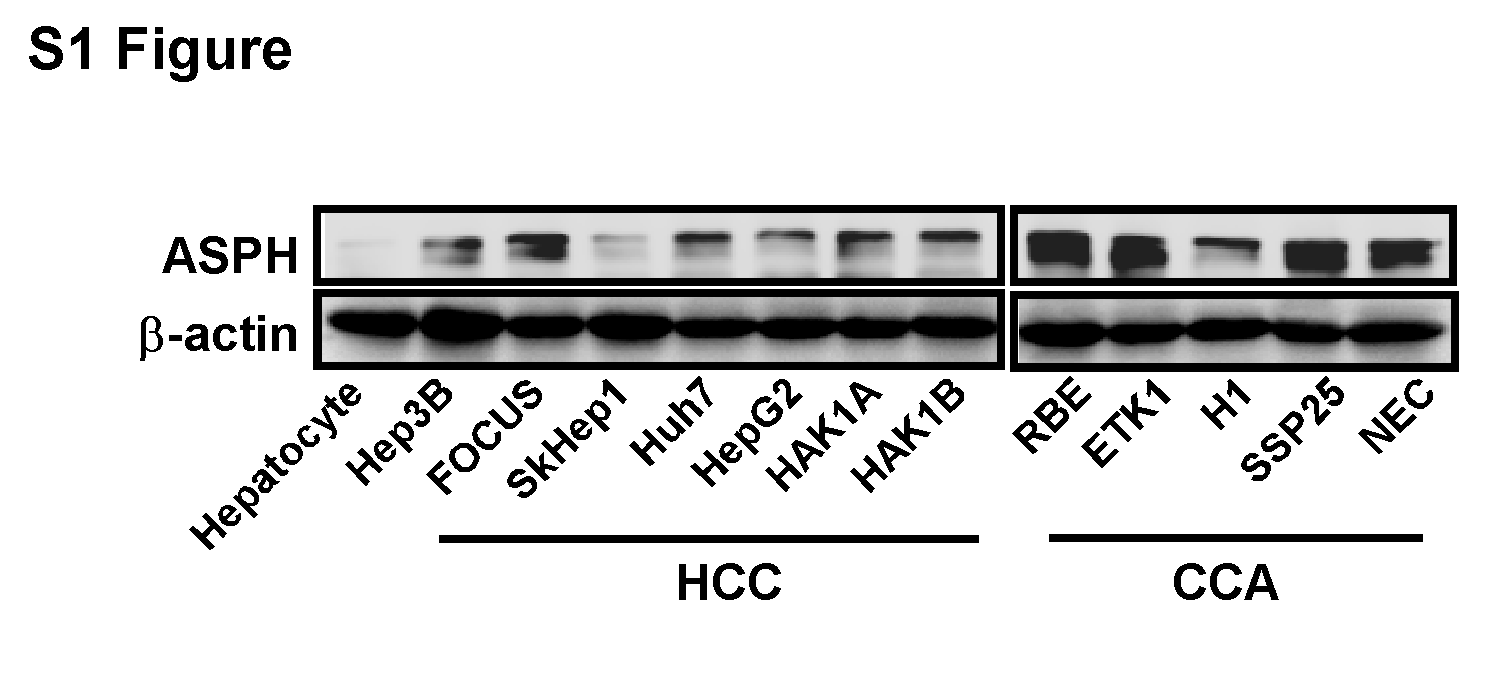

Supplement: S1 Fig — ASPH was undetectable in human hepatocytes. Variable levels of ASPH expression were detected in HCC cell lines. High ASPH expression was observed in all 5 CCA cell lines. β-actin served as the protein loading control. (TIF) [file pone.0150336.s001.tif]

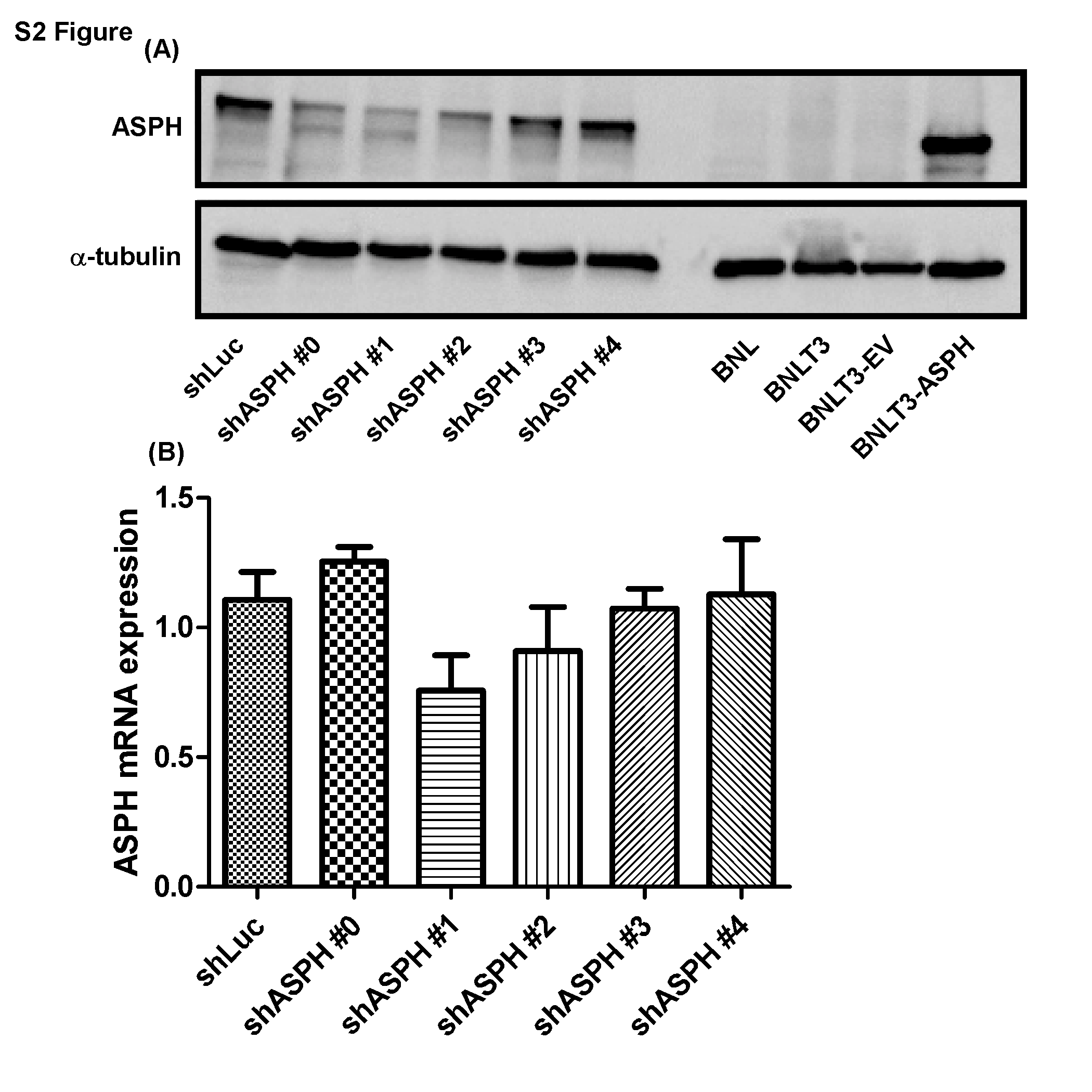

Supplement: S2 Fig — (A) Immunoblotting results of ASPH expression. SSP25 cells were infected with lentivirus containing shRNA-luciferase (shLuc), shRNA-ASPH (shASPH) #0, #1, #2, #3, and #4. (B) Relative ASPH mRNA expression was determined as indicated. (TIF) [file pone.0150336.s002.tif]

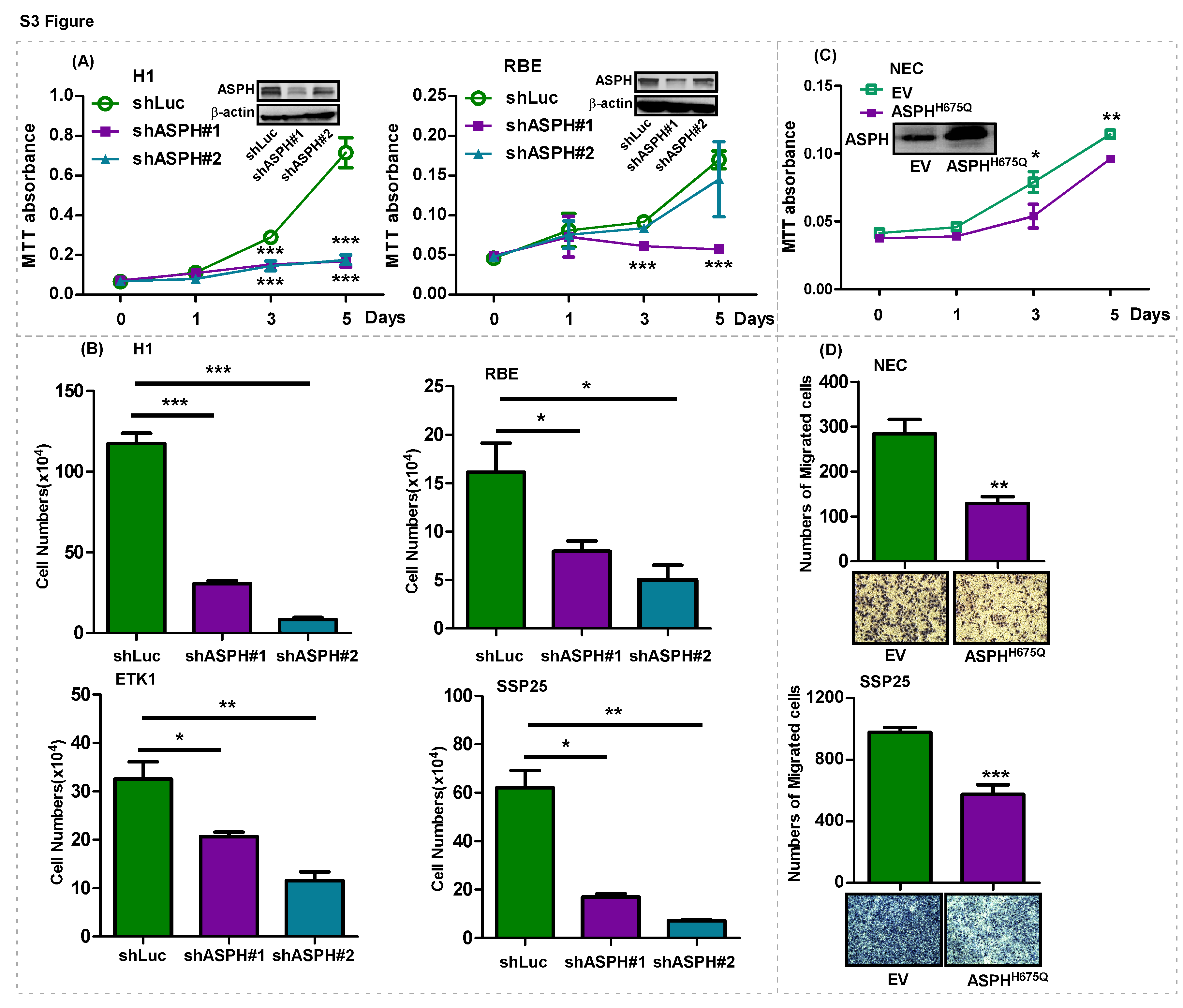

Supplement: S3 Fig — (A) MTT assay results were determined at days 0, 1, 3, and 5 in H1 and in RBE cells infected with lentivirus containing shLuc or shASPH. (B) Cell proliferation as determined by cell counting for H1, RBE, ETK1, and SSP25 CCA cells infected with lentivirus containing shLuc or shASPH. (C) MTT assay results were measured in NEC cells transfected with empty vector (EV) or mutant ASPHH675Q with 80% reduced enzymatic activity. (D) Migrated cell numbers were measured in NEC and SSP25 cells transfected with EV or ASPHH675Q. ***, p-value <0.001; **, p-value <0.01; *, p-value <0.05. (TIF) [file pone.0150336.s003.tif]

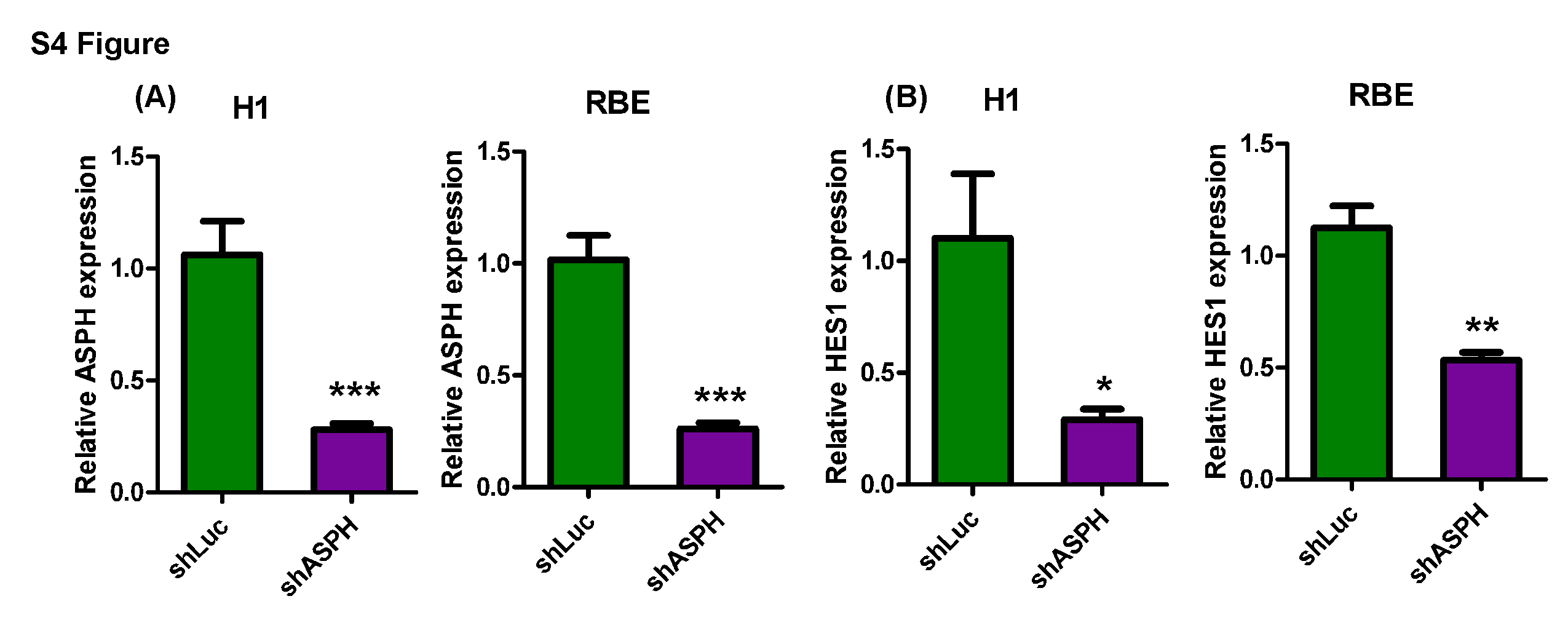

Supplement: S4 Fig — (A) Relative ASPH mRNA expression was determined in H1 and RBE CCAs infected with lentivirus containing shLuc or shASPH. (B) Relative HES1 mRNA expression was shown as indicated. ***, p-value <0.001; **, p-value <0.01; *, p-value <0.05. (TIF) [file pone.0150336.s004.tif]

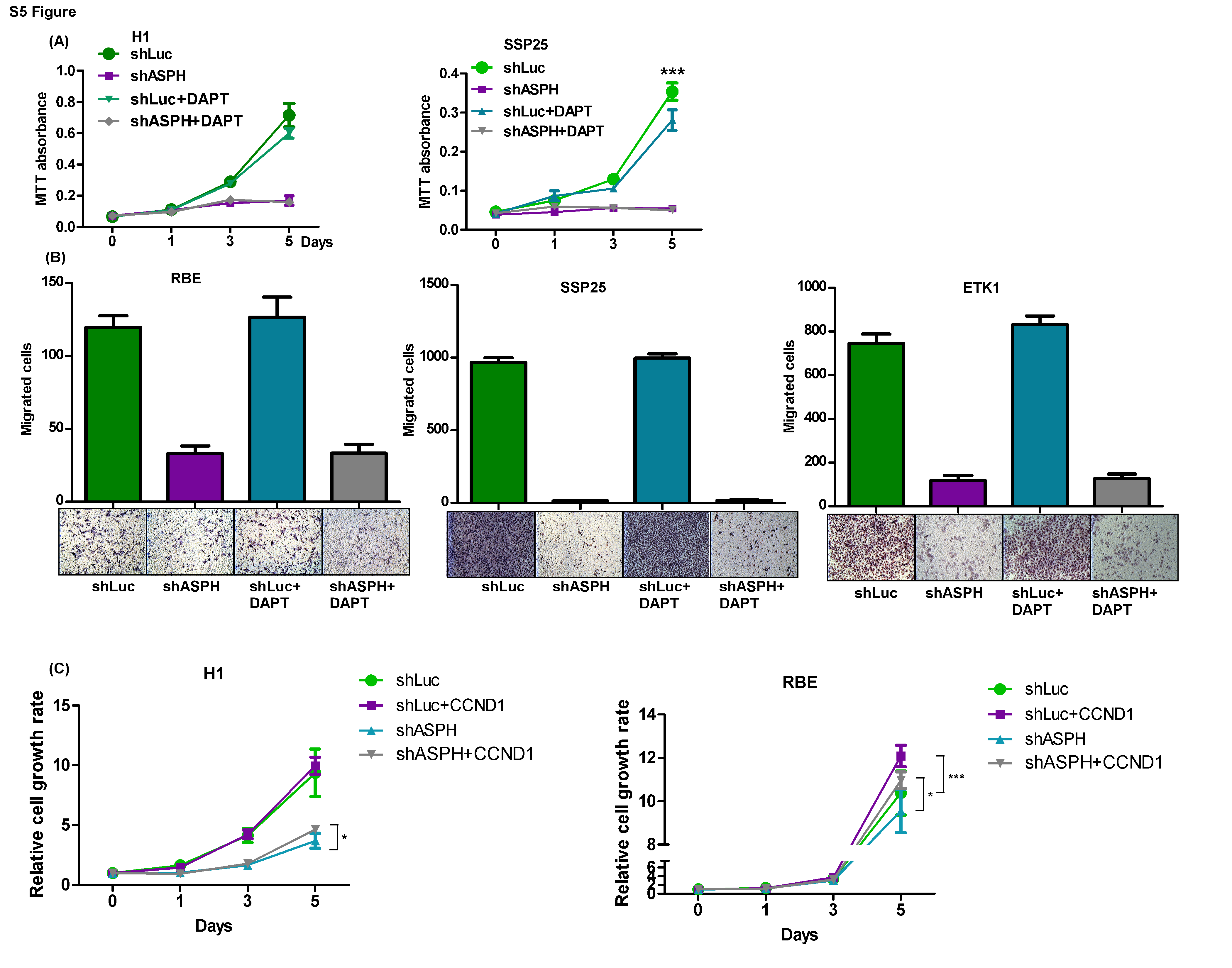

Supplement: S5 Fig — (A) MTT assay results obtained in H1 and SSP25 cells with shLuc or shASPH in the presence or absence of 10 μM γ-Secretase inhibitor (DAPT). (B) Migrated cell numbers were measured in RBE, SSP25, and ETK1 cells as indicated. DAPT had little effect on CCA viability and migration. (C) Relative cell growth rate was determined in H1-shLuc, H1-shASPH, RBE-shLuc, and RBE-shASPH in the presence or absence of cyclin D1 (CCND1) as indicated. *, p-value <0.05; ***, p-value <0.001. (TIF) [file pone.0150336.s005.tif]

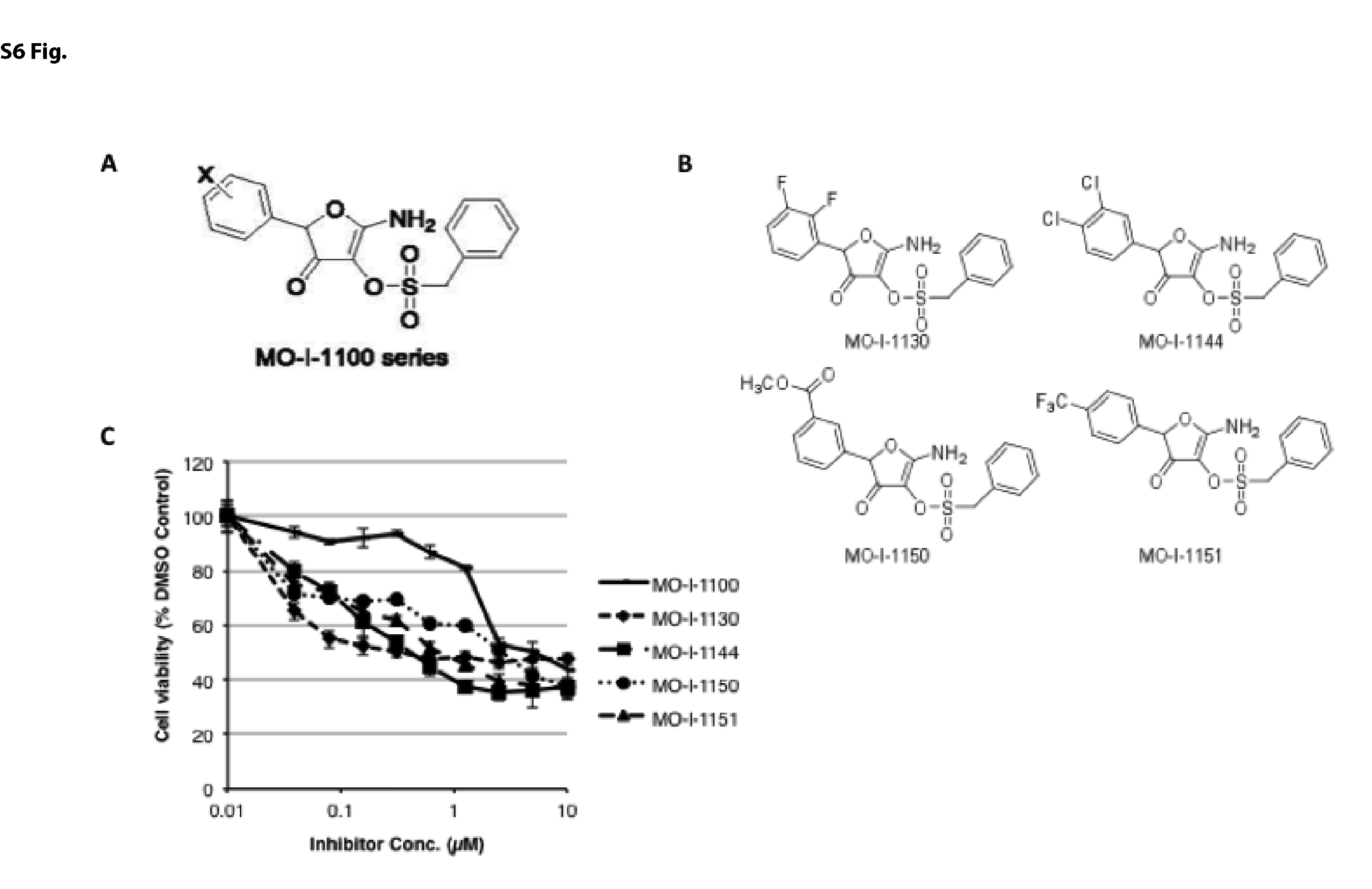

Supplement: S6 Fig — (A) Structure of the 2nd generation SMI compound MO-I-1151. (B) Structures of representative 2nd generation SMIs of β-hydroxylase activity. (C) An analysis of their effect on cell viability. 2nd generation SMIs were 10–50 times more potent than the parent MO-I-1100 compound and MO-I-1151 was selected for further studies based on its strong inhibitory effect. (TIF) [file pone.0150336.s006.tif]

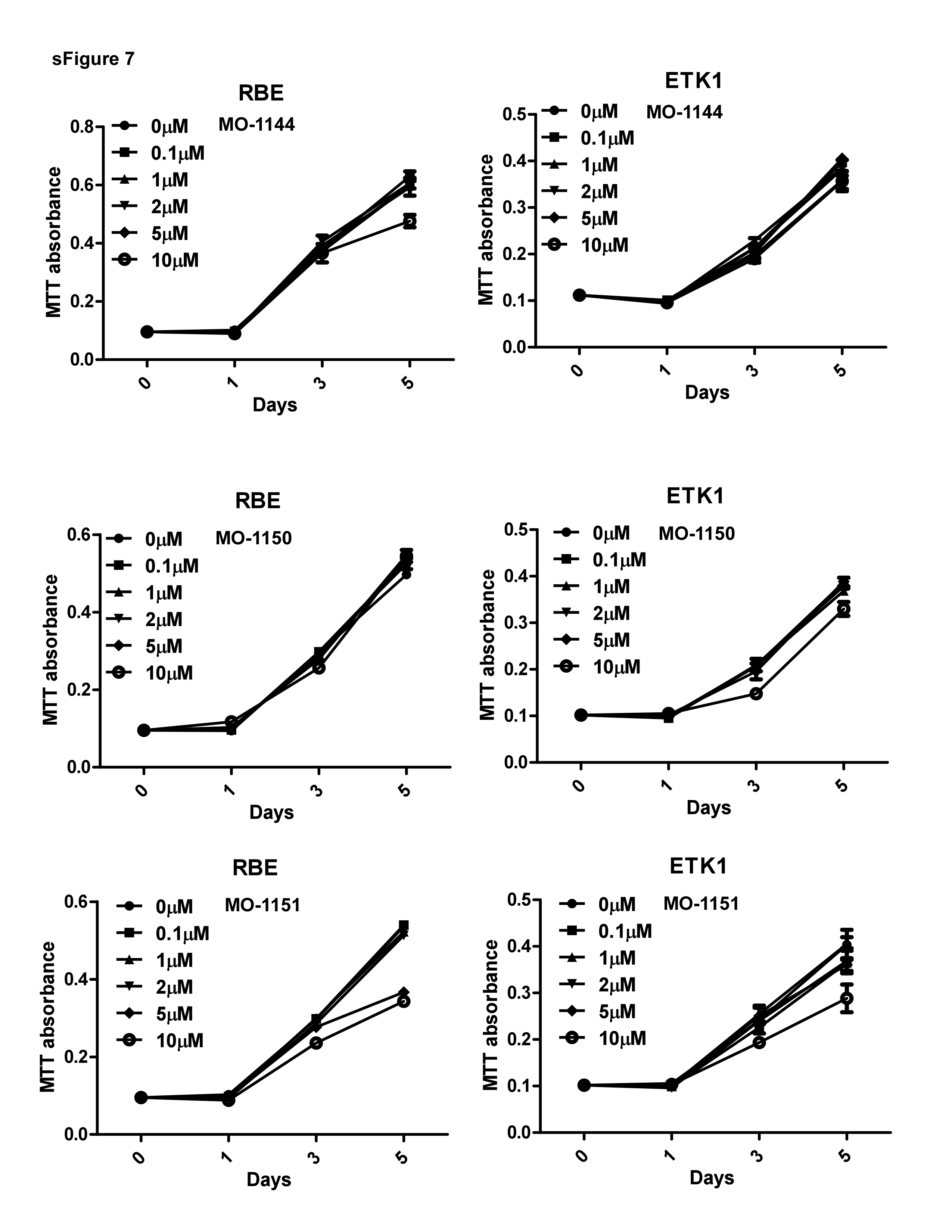

Supplement: S7 Fig — Relative cell growth curves were determined in RBE and ETK1 cells in the presence of MO-I-1144, MO-I-1150, and MO-I-1151, respectively, at the indicated concentrations. MO-I-1151 was found to be the most active and potent. (TIF) [file pone.0150336.s007.tif]

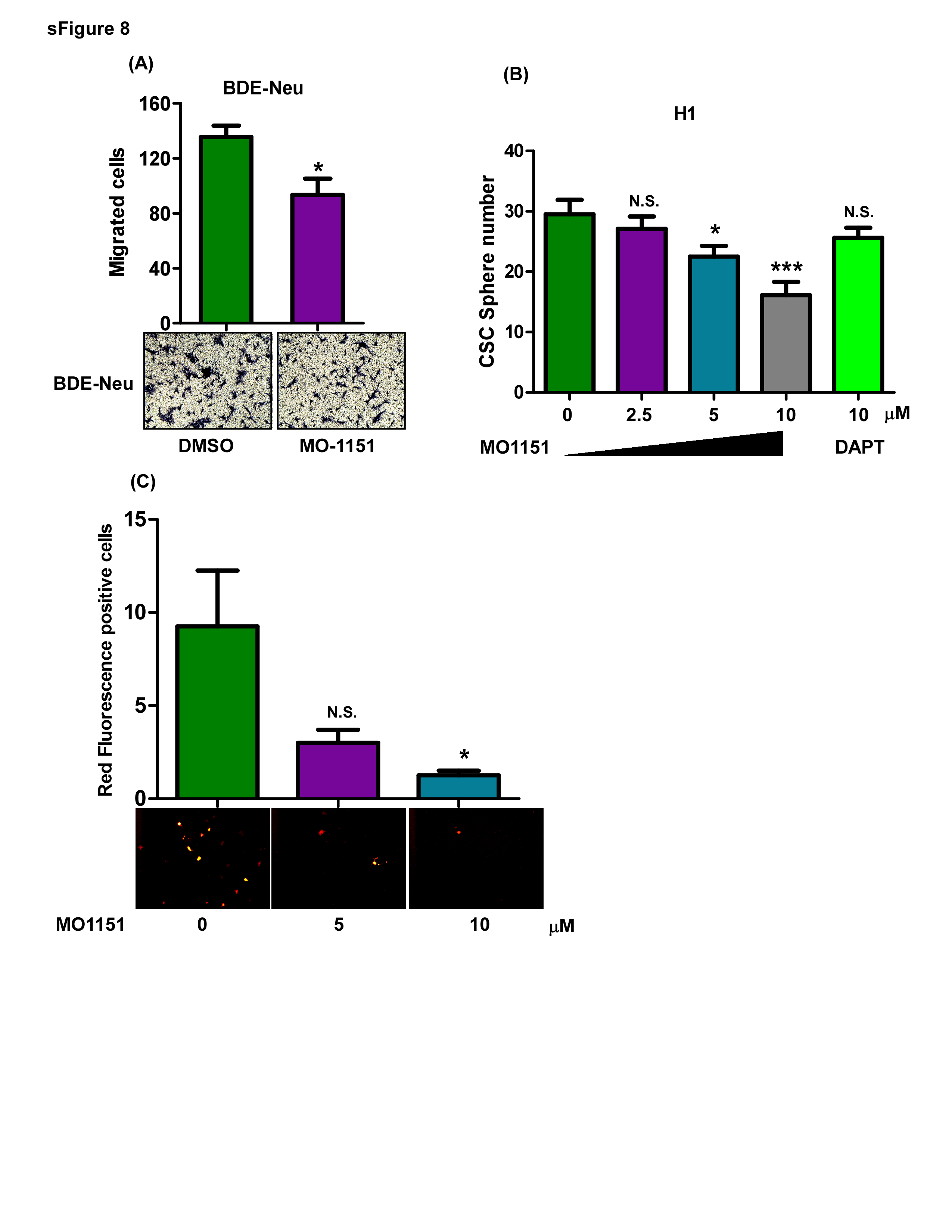

Supplement: S8 Fig — (A) Migrated cell numbers were measured in BDE-Neu cells treated with DMSO or 10 μM MO-I-1151. (B) CSC sphere numbers were determined in H1 cells treated with MO-I-1151 at indicated concentrations or 10 μM DAPT. (C) 12XCSL-DsRedExpressDL (notch reporter) was transfected in RBE CCA cells and then the cells were treated with MO-I-1151 as indicated concentrations. Red fluorescence positive cells were quantified as an index for notch transcriptional activity. ***, p <0.001; *, p <0.05. (TIF) [file pone.0150336.s008.tif]

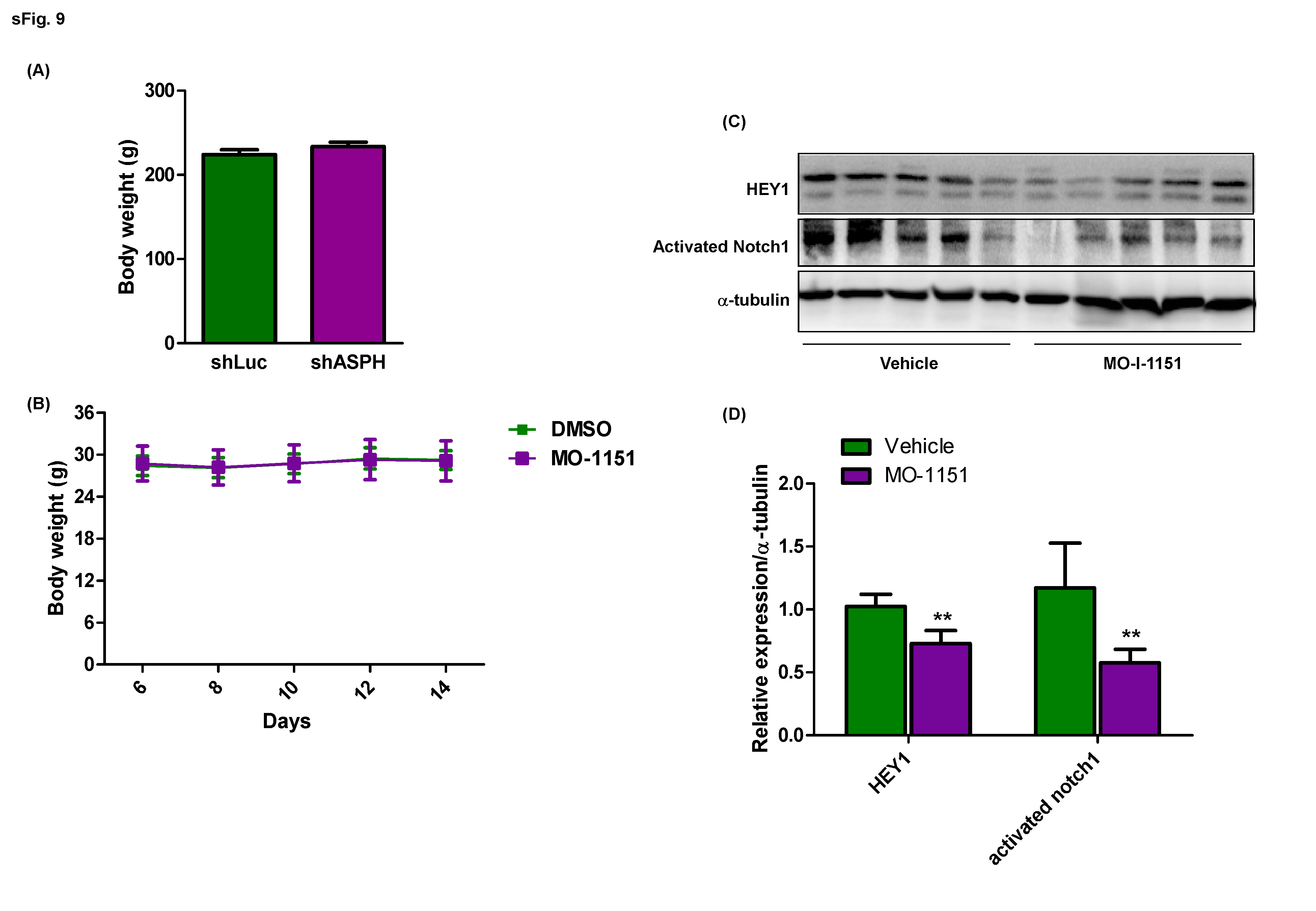

Supplement: S9 Fig — (A) The body weight of rats inoculated with BDE-Neu-shLuc and BDE-Neu-shASPH. (B) The body weight of the mice subcutaneously injected with H1 cells and challenged with vehicle (DMSO) or MO-I-1151 showing no change over 14 days. (C) Immunoblot results of activated Notch1, Hey1, and α-tubulin were determined in H1 tumors (n = 5) treated with vehicle (DMSO) or MO-I-1151. (D) Densitometry of the immunoblots (*p<0.05; **p<0.01). (TIF) [file pone.0150336.s009.tif]
